# An asymptomatic detachment of the appendix evolved to giant abscess and complete colliquative necrosis: pivotal role of computed tomography in patient management

**DOI:** 10.1259/bjrcr.20200125

**Published:** 2020-12-11

**Authors:** Anna Olga Di Vincenzo, Anna Parmeggiani, Mario Casavola, Stefano Leonardo, Rita Golfieri

**Affiliations:** 1 Department of Radiology, Azienda Ospedaliero-Universitaria di Bologna, Bologna, Italy; 2 Department of Radiology, Azienda USL di Imola, Santa Maria della Scaletta Hospital, Imola, Italy

## Abstract

Acute appendicitis (AA) is one of the most common causes of acute abdominal pain and it generally affects young males in the second or third decade of their life. Due to its often insidious presentations, the diagnosis is challenging and, if delayed, can lead to life-threatening complications. This report describes a rare case of an almost asymptomatic complicated appendicitis caused by an appendicolith followed by spontaneous detachment of the vermiform appendix and its complete colliquative necrosis with abscess formation. Thus far this is the first case of spontaneous appendix avulsion in an adult where the appendix is entirely colliquated into an abscess.

## Clinical presentation

A 33-year-old Caucasian male presented himself to the emergency department with severe lower left-side abdominal pain. He complained of abdominal discomfort for the previous 10 days, with associated bloodless mucous diarrhoea. He mentioned having taken rifaximin without any benefit for four days with a light diet associated, on suspicion of gastrointestinal infection. His past medical history was unremarkable.

On physical examination, performed in the emergency room, the abdomen was treatable, widely painful, with no signs of acute abdomen or peritoneal involvement. The patient appeared ill with a heart rate of 93/min, blood pressure of 114/67 mm Hg and body temperature of 36.8°C.

Laboratory tests showed leukocytosis with a WBC count of 19.11/µL, of which 82.7% were neutrophils, haemoglobin at 12.2 g dl^−1^, C-reactive protein of 20.42 mg dl^−1^ (normal range <0.50 mg dl^−1^) and serum creatinine of 6.56 mg dl^−1^ (normal range 0.5–1.0 mg dl^−1^). All the other blood chemical tests were within the normal range.

## Investigations/Imaging findings

The patient underwent an ultrasound (US) examination of the abdomen, which revealed the presence of a conglomerate of intestinal loops with thickened and edematous walls and a surrounding hypo-echogenic area whose nature was difficult to define in the right iliac fossa, considering it in the first hypothesis as a fluid collection.

**Figure 1.**
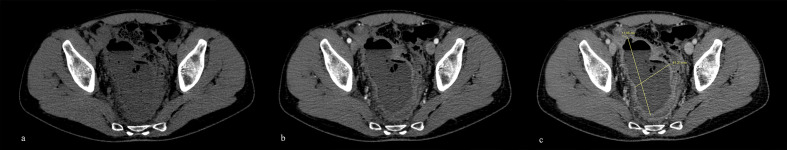
Axial pre-contrast (a) and post-contrast CT scan in arterial phase (b) and portal venous phase (c) shows a 11.86×6.1 cm inhomogeneous pelvic mass with discreet wall contrast enhancement.

**Figure 2.**
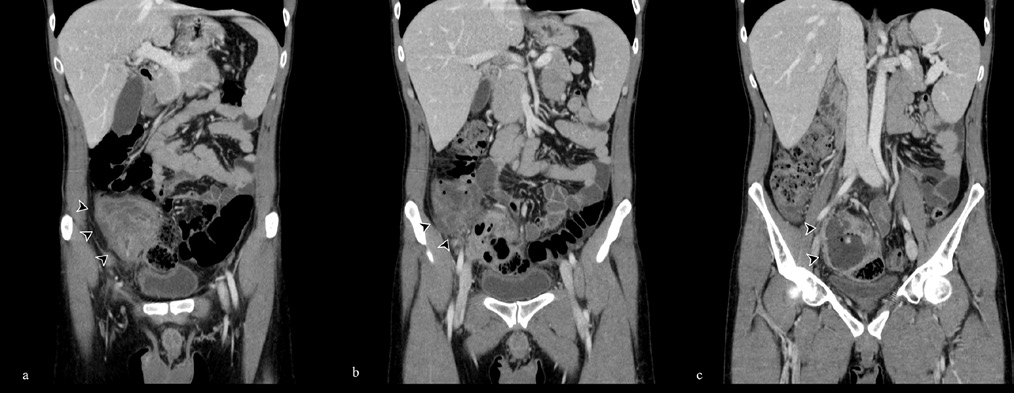
(a–c) Coronal post-contrast CT scan reconstructions in portal venous phase show the inhomogeneous abdominal-pelvic mass with discreet wall contrast enhancement (arrowheads).

**Figure 3.**
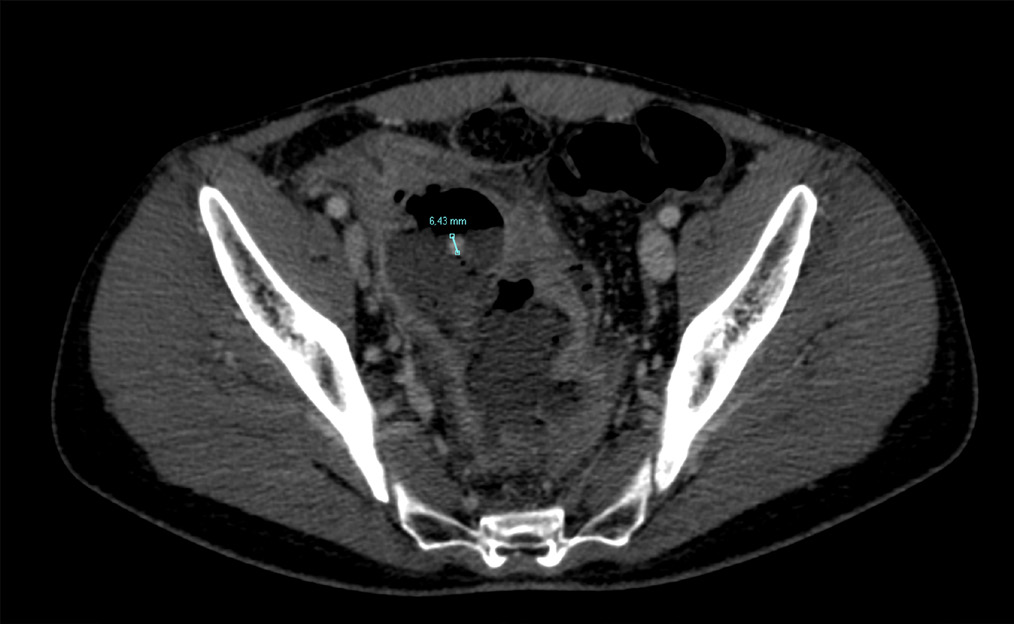
Axial post-contrast CT scan in portal venous phase shows a subcentimetric appendicolith inside the abscess.

**Figure 4.**
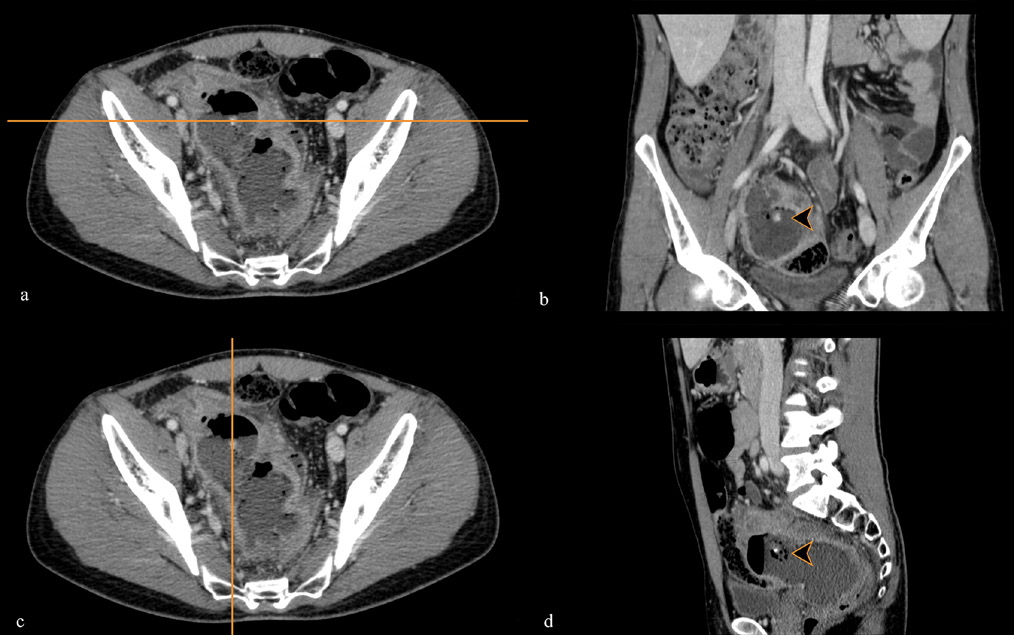
Post-contrast CT scan in portal venous phase: Multiplanar Reconstruction (MPR) images obtained at the level of the subcentimetric appendicolith (arrowheads) inside the abscess along the axial plane (a, c), coronal plane (b) and sagittal plane (d).

**Figure 5.**
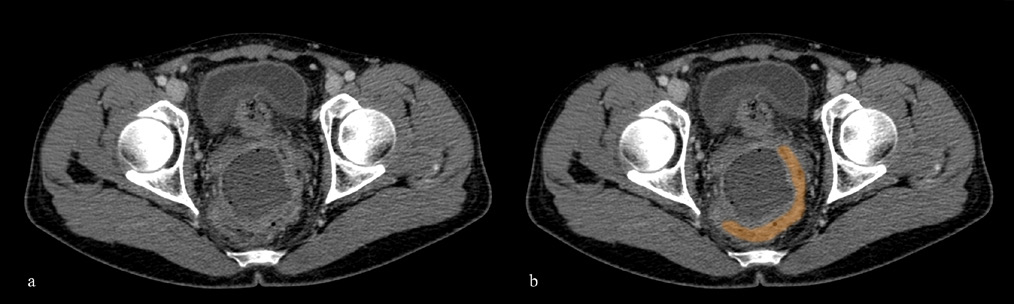
(a) Axial post-contrast CT scan in portal venous phase shows the sigmarectum (highlighted area in b) compressed and dislocated by the abscess in left iliac fossa.

Due to the ambiguous finding, a computed tomography (CT) exam with contrast medium (CM) was required, following an adequate volume expansion of the patient in order to prevent post contrast acute kidney injury (PC-AKI) considering the high serum creatinine value. The investigation showed an uneven collection of fluid material of 11.86×6.1 cm, measured in the axial plane, (Figures 1 and 2 ) extended into the lower right abdomen and pelvis, whose wall underwent enhancement after the administration of CM, with gaseous coefficients and an irregular calcification of 6.4 mm in diameter inside (Figures 3 and 4). In this context, it was not possible to appreciate neither the vermiform appendix nor the last ileal loop, with the exception of some loops of the small intestine, compressed and endowed with mural hyperenhancement. Furthermore, the fluid collection compressed the sigma-rectum, displacing it and making it difficult to be recognized (Figure 5). Only the distal third of the rectal ampoule was detectable. Some enlarged lymph nodes (maximum diameter of 1.1 cm) were identified in the right iliac fossa in absence of free peritoneal liquid.

In order to exclude the clinical suspicion of a rectosigmoid neoplasm, due to mucorrhea, abdominal pain and changes in bowel habits, a proctosigmoidoscopy has also been performed. The investigation had a negative result: the mucosa was normal, while the proximal rectum showed a tumefaction compatible with ab-extrinsic compression.

## Treatment

Considering these findings, the patient underwent an urgent surgical intervention on the same day. The operation started laparoscopically and it was converted to laparotomy due to the difficult release of the anatomical structures in the context of an extensive abscess. Firstly, the last ileal loop has been released, filled with pus and with signs of bowel perforation; secondly the cecum and the appendix were released, this last was detached and completely necrotic into the abscess. Then, an ileocecal resection has been performed and the surgical specimen has been sent for histological and microbiological examination, which confirmed the presence of a massive necrotizing peritoneal abscess, rich in fibrin and granulocytes, with intense oedema and inflammation of the ileocecal walls.

The appendix was completely colliquated within the abscess, to the point that just some fragments of its walls have been found. In these fragments, the necrosis had caused the loss of the mucous membrane and the muscular tunic was the only identifiable layer.

Moreover, the absence of granulomas excluded a chronic inflammatory bowel disease as the possible cause of the inflammatory process.

## Outcome

There were no postoperative complications and three weeks after discharge the patient was asymptomatic with normal white blood cell count.

## Discussion

Acute appendicitis (AA) is one of the most common causes of acute abdominal pain and occurs generally in males between the age of 10 and 30; appendectomy is one of the most commonly performed surgical procedures worldwide. The peak incidence usually occurs in the second or third decade of life and the disease is less common at both extremes of age.^1,2^

It is the most common non-obstetric surgical emergency during pregnancy, with an incidence of 6.3 per 10 000 pregnancies during the antepartum period (compared with 9.6 per 10 000 in non-pregnant persons) and increasing to 9.9 per 10 000 postpartum.^3^ In Italy, from 2001 to 2013, the incidence of AA decreased by 4.7% per year, with a peak incidence of 146 in 2001 per 100 000 inhabitants.^4^

The cause of AA is usually an obstruction of the appendiceal lumen. This can be related to lymphoid hyperplasia, an appendicolith (stone of the appendix), impacted stool or some other mechanical etiologies. Appendiceal tumours such as carcinoid tumours, intestinal parasites and hypertrophied lymphatic tissues are all known as other causes of appendiceal obstruction and AA. Nevertheless, in some cases, the exact aetiology of AA remains unknown.

The obstruction leads to small vessels occlusion and lymphatic stasis, distention, bacterial overgrowth (common organisms include Escherichia coli, Peptostreptococcus, Bacteroides and Pseudomonas), ischaemia and acute inflammation. If not promptly treated, it can lead to necrosis, gangrene and perforation with the consequent risk of peritonitis, which is burdened by a high morbidity.^5^ Recent theories focus on genetic factors, environmental influences, and infections; although no defined gene has been identified, the risk of AA is roughly three times higher in members of families with a positive history of appendicitis than in those with no family history.^6^

Therefore, despite numerous aetiological factors having been recognized, the most widely reported and accepted mechanism is the obstruction of the appendiceal lumen by an appendicolith**,** since peristaltic action is usually insufficient to expel it into the cecum, leading to the inflammatory state. Moreover, the presence of an appendicolith has been correlated with earlier and higher rates of appendicular perforation, so much that this finding automatically defines appendicitis as complicated, excluding its conservative treatment in advance.^7^ In addition, a study conducted by Ishiyama et al.^8^, has proved that the presence of an appendicolith with a diameter greater than 5 mm and located at the base of the appendix is significantly associated with gangrenous appendicitis. This finds confirmation even in our case, since the presence of an appendicolith of 6.4 mm in diameter, identified as a high density formation on CT, could have given rise to appendicular occlusion which resulted in abscess, gangrene and detachment of the appendix.

To the best of our knowledge, even if in literature have already been described some cases of AA caused by an appendicolith and one case of an appendicular abscess evolved in detachment of the appendix, our report is the first one of an appendicular abscess of such conspicuous dimensions evolved to complete colliquative mucosal necrosis of the vermiform appendix, whose fragments were found only at the histopathological examination.^9,10^

Traditionally, the classification of the severity level of AA has an important value for the management and the prognosis. In fact, AA presents a spectrum varying from uncomplicated (acute suppurative) to moderately severe (acute gangrenous) to severe (perforated with abscess).^11^ The uncomplicated form includes phlegmonous inflammation while the complicated one involves gangrenous inflammation, perforation with eventual local/diffuse peritonitis or periappendicular abscess formation. This distinction has a significant consequence in the management of AA: in the uncomplicated form, antibiotic treatment can be a successful therapeutic option, while the complicated one requires surgical intervention.

Nevertheless, this classification still presents some issues, as it is currently still unclear whether the distinction should be built on the histological findings or on the perioperative valuation.^12^ It is important to point out that, even if in literature no agreement regarding the most preferable approach for acute uncomplicated appendicitis has yet been reached, appendectomy (both laparotomic and laparoscopic) is still the most common treatment.^13^

Despite its frequency, the diagnosis of AA continues being challenging because of the absence of clinical signs or positive blood results in 55% of the cases and the number of missed diagnoses ranges between 20 and 40%.^14^

Classical presentation is characterized by right lower quadrant (RLQ) pain, fever, elevated white blood cell (WBC) count, loss of appetite, nausea, and vomiting. In 60% of the cases, pain starts in the umbilical region and then migrates to the RLQ.^15^ In order to reach a confident diagnosis, more than 10 different clinical scores have been proposed and, among these, Alvarado and RIPASA are currently the two most widely used. Alvarado scoring system assigns one or two points to eight different clinical signs and laboratory tests with a total maximum possible score of 10, while RIPASA is comprised in 15 parameters (demographic, clinical and laboratory based); to each of those parameters the aforementioned scoring system assigns from 0.5, one or two points reaching a maximum possible score of 15. The cut-off value considered as positive for AA is 7 for the Alvarado system and 7.5 for RIPASA. As revealed in a recent meta-analysis by Frountzas et al^16^, RIPASA presents higher sensitivity and lower specificity if compared with the Alvarado one. This evidence seems to suggest that the best clinical choice to provide an accurate diagnosis could be the use of both, at least during the first approach.

In our patient, a score of 3 has been calculated with the Alvarado score system and a score of 3.5 with the RIPASA one, both not suggestive of AA.

This result confirms that, although the diagnosis of appendicitis is primarily medical, clinical scores alone may be insufficient, especially in the case of an atypical presentation (most common in elderly patients or pregnant females), leading to unsuccessful identification and delayed treatment. This can lead to complications like appendiceal perforation and, in 20% of the cases, to subsequent phlegmon (an ill-defined mass of inflammatory tissue) or abscess formation (a well-delineated, walled-off fluid collection) with high risk of peritonitis, possibly resulting in the patient’s death.^15^

Consequently, it is common practice to integrate clinical suspicion with the contribution of imaging, since both US and CT are valid tools for rapid diagnosis and better assessment of the appendicitis severity. In fact, CT scans are able to identify those radiological features indicative of complicated appendicitis, such as abscess, appendicolith, perforation or suspicion of a tumour, in order to ensure the earliest possible surgical treatment.^17^ Although US has lower sensitivity and specificity (reported to be between 71 and 97%) than CT (between 83 and 98%), it is most indicated for children and pregnant females in order to avoid ionizing radiation, as claimed by the WSES Jerusalem guidelines.^14,18^

What has previously been reported finds validation in our experience, since our patient had laboratory values suggestive of an ongoing inflammatory state, but he did not present a typical AA symptomatology.

Therefore, only the contribution of imaging allowed to identify the abdominal fluid collection.

On the other hand, the final confirmation of appendiceal abscess diagnosis was achieved by the histological exam, essential to specifically exclude the co-presence of other pathologies that could have induced AA and to demonstrate the actual etiopathogenesis of the process.

Furthermore, the detection of appendicular fragments with colliquative necrotic appearance at the histological examination explains why it has not been possible to identify the whole appendix into the fluid collection in the CT investigation; consequently, being the fragments deprived of vascular supply, there was no enhancement after CM administration that could have been useful for their identification.

Nonetheless, despite the continuous advancement of diagnostic tools, the AA remains an insidious pathology and a challenging diagnosis, especially in atypical cases.

In conclusion, although a spontaneous detachment of the appendix with an abscess as an aetiology of lower left abdominal pain is a rare entity, it ought to be considered in the differential diagnosis when approaching a patient who presents laboratory results suggestive of appendicitis and, in order to achieve a more accurate diagnosis, CT imaging should be performed prior to the intervention.

## Learning points

1. The diagnostic hypothesis of appendicitis must be considered even in case of atypical paucisymptomatic patients with laboratory tests suggestive of inflammatory condition.
2. Compared to the US examination, the CT scan has a pivotal role in the assessment of patients with atypical symptoms of appendicitis and it is also able to lead to the correct management.
3. In case of detachment, the appendix could not be visualized neither in the US nor in the CT imaging due to the colliquative mucosal necrosis and it was possible to detected fragments only during the histological examination.
